# Comments on “Right ventricular failure in septic shock: characterization, incidence and impact on fluid responsiveness”: which parameter to assess right ventricular failure and venous congestion?

**DOI:** 10.1186/s13054-021-03473-0

**Published:** 2021-04-09

**Authors:** Osama Abou-Arab, Mouhamed D. Moussa, Christophe Beyls, Yazine Mahjoub

**Affiliations:** 1 Department of Anesthesiology and Critical Care Medicine, Amiens Picardie University Hospital, 1 Rue du Professeur Christian Cabrol, 80054 Amiens, France; 2 Department of Anesthesiology and Critical Care Medicine, Institut, Cœur – Poumon, CHU Lille, 2 Avenue Oscar Lambret, 59037 Lille, France

To the editor

We read with great interest the article by Veillard Baron et al. about right ventricular (RV) failure in septic shock and its link with fluid responsiveness [1].

The authors defined right ventricular failure as the association of RV dilatation (RV/LVEDA < 0.6) and increased central venous pressure (CVP ≥ 8 mmHg). They showed that this definition of RV failure is associated with lack of fluid responsiveness. Therefore, CVP could be used as an additional measurement to RV dilatation to discriminate between patients with and without congestive RV failure. This rationale seems attractive. However, several limitations may weaken the conclusion of this study.

## Parameters used to define RV failure

The echocardiographic pattern chosen by the authors is a limited RV dilation that may be an adaptive response (as shown during high intensity or endurance exercise) without actual RV failure. In two-dimensional echocardiography, the consensus definition of the American Society of Echocardiography and the European Association of Cardiovascular Imaging of RV global systolic dysfunction is still based on RV-fractional area contraction (RV-FAC), whilst global RV function is based on right ventricular index of myocardial performance [2]. These parameters would have been of great interest in this setting.

## Measurement of CVP

Several confusing factors could mislead in CVP interpretation in this context, especially because a large number of patients in group 3 has a CVP between 8 and 10 mmHg and several patients of group 1 and 2 has a CVP close to 8 mmHg. First, group 3 patients have a relatively high rate of atrial fibrillation (20%): a factor well known to increase CVP values independently from venous congestion [3]. Second, The CVP threshold of 8 mmHg or greater is questionable knowing that mean systemic filling pressure varies from 7 to 10 cmH2O. Hence, we suggest the use of other markers of venous congestion as hepatic or portal venous Doppler [4].

## PLR maneuver and intra-abdominal pressure (IAP)

Regarding the reliability of PLR maneuver to assess fluid responsiveness, it has been shown more than 10 years ago that an IAP over 12 mmHg may induce false negatives [5]. This point has been discussed by Vieillard-Baron et al. in the limitation part of their study. However, because patients in group 3 have higher values of IAP (median of 11 mmHg and interquartile range of 8–14 mmHg) than other groups of patients, the number of false negatives should not be neglected.

## Data Availability

Not applicable.

## Abbreviations

CVP: Central venous pressure
RV: Right ventricle
