# Supplementary material for: Comments on “Right ventricular failure in septic shock: characterization, incidence and impact on fluid responsiveness”: which parameter to assess right ventricular failure and venous congestion?
Source: Crit Care. 2021 Apr 9;25:136. doi: 10.1186/s13054-021-03473-0 (PMC8034069; doi:10.1186/s13054-021-03473-0)
Supplement: Related texts

# Reply: Authors’ response

**Authors:** Antoine Vieillard-Baron, Guillaume Geri

**Affiliations:** 3 grid.50550.35 0000 0001 2175 4109 Intensive Care Unit, University Hospital Ambroise Paré, Assistance Publique des Hôpitaux de Paris, 92100 Boulogne-Billancourt, France; 4 grid.460789.4 0000 0004 4910 6535 UFR de Médecine Simone Veil, Université Paris Saclay, 78000 Versailles, France

Dear Dr Abou-Arab,

We acknowledge that definition of right ventricular (RV) failure in our study is far from perfect. The key message is that patients with RV failure could not be fluid-responsiveness despite significant pulse pressure variations.

Distinction between RV dysfunction and failure is not easy to draw, while crucial in the critically ill patients. Lahm et al. re-emphasized that RV systolic dysfunction indicates structural RV changes, which could cause in the most severe form RV failure [6]. We reported the tricuspid annular systolic excursion (TAPSE) was similar whatever CVP and RV size and did not classify patients in the RV failure group. Such surprising results could be explained because parameters of RV systolic function, as TAPSE and fractional area contraction, did not tightly reflect coupling between the right ventricle and the pulmonary circulation [7].

From a physiological point of view, RV failure is defined as the association of RV dilatation with systemic congestion and may even occur when the cardiac output is still maintained [6, 8]. Moderate or even mild RV dilatation cannot be anymore an adaptation when associated with systemic congestion. Magder’s group has reported the ability of healthy individuals to dramatically increase cardiac output during sustained exercise with only a slight increase in right atrial pressure, while patients who previously received heart transplantation had a much lower increase in cardiac output with a significant elevation in right atrial pressure [9].

We agree that the CVP threshold above which systemic congestion may be suspected is still questionable. However, the kidney is known to be very sensitive to any slight alteration in CVP. Chen et al. reported in critically ill patients an increased risk of acute kidney injury for each 1 cmH_2_O increased CVP when compared to the “normal value” which was ≤ 7 cmH_2_O [10]. CVP is also known to be much more associated with worsening of renal function than a low cardiac index, especially when above 8 mmHg. Normal value of mean systemic filling pressure (MSFP) was indeed reported around 7–10 mmHg in animals. How it may be translated to critically-ill patients remains questionable and we reported a MSFP around 13 mmHg in septic patients just after death [11].

Finally, we used passive leg raising to assess fluid-responsiveness, while Mahjoub et al. reported false-negative may be indeed observed in case of elevated intra-abdominal pressure. But what the authors didn’t say is that it was mainly observed in case of pressure ≥ 16 mmHg, a situation rarely observed in our study.

In conclusion, our study evaluated a new definition of RV failure, associating RV size evaluated by echocardiography and CVP. Future works are required to confirm our approach and to improve detection and characterization of RV failure in critically-ill patients.
